# Computer-aided autism diagnosis using visual attention models and eye-tracking: replication and improvement proposal

**DOI:** 10.1186/s12911-023-02389-9

**Published:** 2023-12-14

**Authors:** Felipe O. Franco, Jessica S. Oliveira, Joana Portolese, Fernando M. Sumiya, Andréia F. Silva, Ariane Machado-Lima, Fatima L.S. Nunes, Helena Brentani

**Affiliations:** 1 Interunit PostGraduate Program on Bioinformatics, Institute of Mathematics and Statistics (IME), University of São Paulo (USP), 05508-090 São Paulo, SP Brazil; 2 Department of Psychiatry, University of São Paulo’s School of Medicine (FMUSP), 05403-903 São Paulo-SP, Brazil; 3 School of Arts, Sciences and Humanities (EACH), University of São Paulo (USP), 03828-000 São Paulo-SP, Brazil

**Keywords:** Autism spectrum disorder, Eye-tracking, Machine learning, Classifier, Replicability

## Abstract

**Background:**

Autism Spectrum Disorder (ASD) diagnosis can be aided by approaches based on eye-tracking signals. Recently, the feasibility of building Visual Attention Models (VAMs) from features extracted from visual stimuli and their use for classifying cases and controls has been demonstrated using Neural Networks and Support Vector Machines. The present work has three aims: 1) to evaluate whether the trained classifier from the previous study was generalist enough to classify new samples with a new stimulus; 2) to replicate the previously approach to train a new classifier with a new dataset; 3) to evaluate the performance of classifiers obtained by a new classification algorithm (Random Forest) using the previous and the current datasets.

**Methods:**

The previously approach was replicated with a new stimulus and new sample, 44 from the Typical Development group and 33 from the ASD group. After the replication, Random Forest classifier was tested to substitute Neural Networks algorithm.

**Results:**

The test with the trained classifier reached an AUC of 0.56, suggesting that the trained classifier requires retraining of the VAMs when changing the stimulus. The replication results reached an AUC of 0.71, indicating the potential of generalization of the approach for aiding ASD diagnosis, as long as the stimulus is similar to the originally proposed. The results achieved with Random Forest were superior to those achieved with the original approach, with an average AUC of 0.95 for the previous dataset and 0.74 for the new dataset.

**Conclusion:**

In summary, the results of the replication experiment were satisfactory, which suggests the robustness of the approach and the VAM-based approaches feasibility to aid in ASD diagnosis. The proposed method change improved the classification performance. Some limitations are discussed and additional studies are encouraged to test other conditions and scenarios.

**Supplementary Information:**

The online version contains supplementary material available at 10.1186/s12911-023-02389-9.

## Background

Autism Spectrum Disorder (ASD) is a neurodevelopmental disorder characterized by impaired social communication, social interaction, and stereotyped and repetitive behaviors [1, 2]. Several studies have been performed to aid the ASD diagnosis using eye-tracking signals, based on different paradigms, but most of these studies require the prior demarcation of Regions of Interest (ROIs) [3–5]. When we select a ROI, we are not considering the Visual Attention Models (VAMs) knowledge, such as image characteristics, and this could impact the results. Pierce et al., [6] used the GeoPref paradigm and obtained an Area Under the ROC Curve (AUC) of 0.71 in classifying Typical Development (TD) and ASD. Moore et. al., [7] hypothesized that more complex social scenes would increase the discrimination between TD and ASD, and proposed the Complex GeoPref. However, they did not observe an improvement in the classification and pointed out that one of the possible reasons is that they had not considered differences in low-level visual properties, such as color and contrast. VAMs have obtained relevance to better understand ASD once computational approaches can be implemented considering characteristics of the human visual model instead of the ROIs [8]. VAMs allow the exploration of two mechanisms that direct visual attention: the Bottom-Up, guided by pixel-level features, based mainly on intrinsic characteristics of the image; and the Top-Down, which is task-oriented and has semantic information of prior knowledge, related to a context [9]. Wang et al. [10] built saliency maps considering three-level features: pixel-level (e.g. color), object-level (e.g. shape), and semantic-level (e.g. faces) features. Their results showed that semantic features were relevant to build these maps in TD and ASD, suggesting Top-Down impairments in ASD. Based on these findings, Oliveira et al. [11] developed an innovative approach considering the three-level features in TD and ASD classification. Two VAMs (TD and ASD) were trained separately to construct saliency maps, which were compared with the individuals’ fixation maps for classification. The average results for AUC, sensitivity, and specificity were 0.82, 0.69, and 0.93, respectively. It is important to note that Oliveira’s results were obtained using stimulus more similar to the Complex GeoPref paradigm, that is, describing scenes of several children interacting, doing yoga, and jumping.

A concern that has gained prominence in the scientific community is the replicability of findings [12] and external validity. As there are different definitions of replicability in different scientific areas, we will use the definition by Patil et al. [13]: replicability is “re-performing the experiment and collecting new data”. In short, replicability involves new data collection and use of similar methods applied on previous studies. Taking this into account, the first contribution of this paper was to verify if the results obtained by the method proposed by Oliveira et al. [11] can be maintained in two different scenarios. In the first scenario, we tested the trained classifier from the previous study to verify the classifier performance using a new stimulus based on the same paradigm without retraining. In this scenario, the TD and ASD VAMs, once trained could be used independently of the stimulus. The second scenario is a replication study that retrains the VAMs using the same parameters previously defined and the same paradigm, but with a new stimulus and a new sample of individuals. Here, the model would be validated but it will be stimulus-dependent. Considering Moore’s study [7], we used a stimulus more similar to the Original GeoPref, that is, with the faces of one child at a time in the center of the screen.

Stimuli based on the visual preference paradigm are widely used in the literature [7, 14]. Their use allows exploring differences in visual attention between TD and ASD, since in ASD there are: greater preference for geometric scenes [7], lower saliency to semantic features [10], and more difficulty in disengaging the gaze (i.e., look away from something in the current focus of attention to attend to something new) [15]. Instead of static photographs, the use of videos can provide a complete set of observations related to eye-tracking but include some challenges to process, which Oliveira et al. [11] have overcome.

Traditionally, VAMs are built with Neural Networks (NN) [16–18], although some studies use Support Vector Machine (SVM) [19, 20]. Given the high performance in classification problems compared to other machine learning algorithms, Random Forest (RF) enjoys special attention [21]. According to a previous query in the main scientific databases, only two studies used methods based on decision trees to build saliency maps to aid in the diagnosis of ASD. Rahman et al. [22] used the XGBoost algorithm, while Startsev et al. [23] used the RF algorithm, but both with small datasets. Therefore, the second contribution was to use RF to train the classifier and evaluate the performance of these new classifiers using the current and previous datasets.

## Material and methods

### Computational model

The original computational method was proposed by Oliveira et al. [11]. VAM learning aims to determine which pixels were fixed by the subjects and which were not. However, each single frame does not have enough fixation points to extract relevant information. In order to solve this problem, Oliveira et al. proposed a preprocessing step for the aggregation of consecutive frames with an average value of motion between them less than 0.33. This threshold was maintained in all experiments, with the exception of the Test of Frame Aggregation Thresholds experiment.

The first step consists of training two VAMs (TD and ASD). These models are built considering features extracted from the pixels that were fixed by the individuals when watching the video, during the eye-tracking process. These data were used to build group-specific saliency maps. The classifier induction algorithms used to build the saliency maps were NN and SVM. The architecture of the NN was composed of ten neurons in a single hidden layer and backpropagation adjustment with Bayesian regularization. For activation functions, they were sigmoid in the hidden layer and linear in the output layer. The stop condition to reach 1000 epochs or error less than 1e-7. The learning rate was 0.01. The other parameters were the default for the “trainbr” function. The SVM Linear classification was performed with default parameters for a small number features from Liblinear [24].

The trained models were used to predict whether a given pixel, represented by its features vector, was fixed or not in a specific group (TD or ASD). To train and test the models, we here applied a 5-fold cross-validation. Each time we used 4 groups of images (80% of images) as the training set and used the remaining group (20% of images) as the testing set.

For the diagnosis of an individual, each evaluated frame contributes to one vote to TD or ASD class according to the similarity between the fixation map of that individual and TD or ASD saliency map. Finally, the individual is classified according to the number of frames classified as TD or ASD. Note that the stimulus used for training the group-specific VAMs is not necessarily the same stimulus used for creation of the saliency maps used for diagnosis. The method was described in detail by Oliveira et al. [11]. The entire process was implemented in MatLab 2015a version 8.5 [25].

### Replication experiment

#### Subjects

Data from 77 new subjects were collected: 44 from the TD group and 33 from the ASD group. All subjects ages ranged from three to 16 years old. The TD subjects were recruited from three public schools and one private school. The ASD subjects were recruited from the Psychiatry Institute, University of São Paulo - School of Medicine. Diagnoses were made based on clinical evaluation by a multidisciplinary team with child psychiatrists, neuropsychologists, and speech therapists, according to the criteria of the Diagnostic and Statistical Manual of Mental Disorders 5 (DSM) [1] and the diagnostic classification of the Autism Diagnostic Observation Schedule (ADOS) 2. Additionally, the Childhood Autism Rating Scale (CARS) was applied for the ASD group, which indicates the ASD severity. The functional cognitive evaluation was performed by a trained neuropsychologist, using Vineland Adaptive Behavior Scales [26]. Intelligence Quotient (IQ) was assessed by Wechsler Intelligence Scale for Children (WISC) [27] or the non-verbal intelligence test *SON-R* 2^1^/_2_-7[a], standardized and validated for the Brazilian population [28], according to the age criteria recommended by the scales. The clinical information of subjects is available in Supplementary Table 1.

#### Apparatus and stimulus

Gaze position signals were collected using a Tobii Pro Fusion [29] recording at 250 Hertz, whereas in the previous study the eye-tracker operated at 300 Hertz, therefore, we had a lower amount of eye-tracking records, with 17% less data.

As in the previous study, a five-point calibration was adopted for each eye-tracking collection. In case of failure, the calibration was repeated, and if a second failure occurred, the subject was excluded from the experiment. The capture software was the Tobii ProLab [30], using the I-VT fixation filter [31]. As in the previous study, an exclusion criterion for eye-tracking data loss of 20% was adopted. This criterion helps to guarantee the quality of data, mainly because children tend to look away when they are no longer interested [32].

**Fig. 1:**
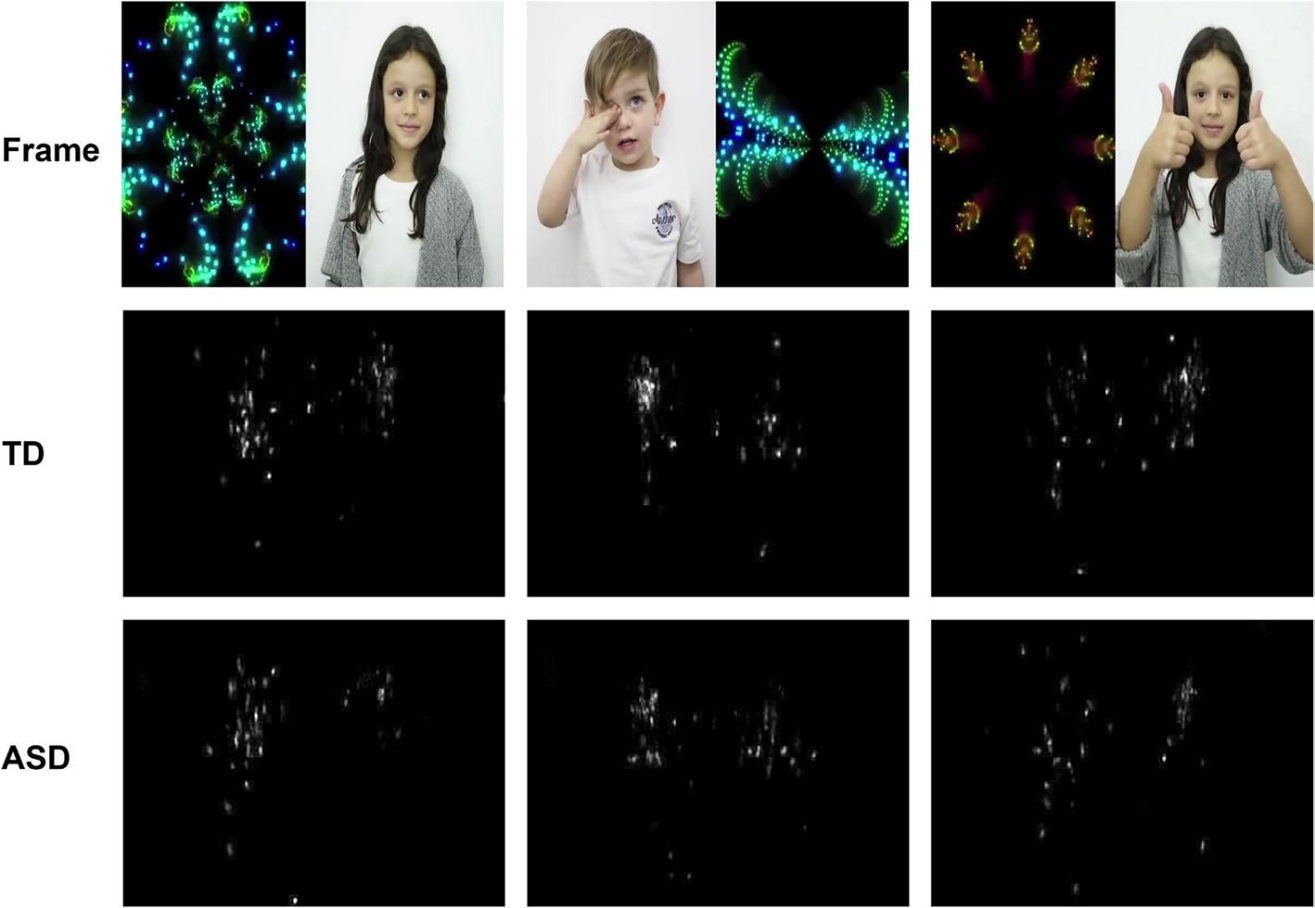
Fixation maps for video frames examples. Abbreviations: TD, Typical Development; ASD, Autism Spectrum Disorder. (This figure was built with XPaint version 2.9.10 [33])

The stimulus consists of a video considering the same paradigm as that used previously in the reference study [11], but with different content. The video is 41-second long, without audio accompaniment, and 30 frames per second presentation. In Supplementary Table 2, this information is listed in comparison to the previous stimulus. Regarding the length of the video, the current video is 22% shorter than that used in the previous study. Figure 1 presents some frames of the stimulus and their respective fixation maps for both groups. A comparison between the features is presented in Table 1.

**Table 1:** Comparison of average values for features of video frames. Values are expressed as average ± standard deviation. The first 13 features correspond to the Steerable Pyramids and have an average value of 1 because there are normalized values, with variation only between pixels

Feature	Description	Previous Dataset	Current Dataset
1	Steerable Pyramid	1.000 ± 0.000	1.000 ± 0.000
2	Steerable Pyramid	1.000 ± 0.000	1.000 ± 0.000
3	Steerable Pyramid	1.000 ± 0.000	1.000 ± 0.000
4	Steerable Pyramid	1.000 ± 0.000	1.000 ± 0.000
5	Steerable Pyramid	1.000 ± 0.000	1.000 ± 0.000
6	Steerable Pyramid	1.000 ± 0.000	1.000 ± 0.000
7	Steerable Pyramid	1.000 ± 0.000	1.000 ± 0.000
8	Steerable Pyramid	1.000 ± 0.000	1.000 ± 0.000
9	Steerable Pyramid	1.000 ± 0.000	1.000 ± 0.000
10	Steerable Pyramid	1.000 ± 0.000	1.000 ± 0.000
11	Steerable Pyramid	1.000 ± 0.000	1.000 ± 0.000
12	Steerable Pyramid	1.000 ± 0.000	1.000 ± 0.000
13	Steerable Pyramid	1.000 ± 0.000	1.000 ± 0.000
14	Itti Color	0.404 ± 0.079	0.160 ± 0.046
15	Intensity	0.770 ± 0.171	0.198 ± 0.052
16	Orientation	0.442 ± 0.073	0.063 ± 0.008
17	Presence of Skin	0.224 ± 0.047	0.166 ± 0.046
18	Color - Red	0.539 ± 0.142	0.468 ± 0.090
19	Color - Green	0.518 ± 0.143	0.458 ± 0.105
20	Color - Blue	0.512 ± 0.156	0.443 ± 0.097
21	Horizon Line	0.503 ± 0.010	0.514 ± 0.003
22	Presence of Face	0.173 ± 0.094	0.074 ± 0.073
23	Presence of People	0.006 ± 0.008	0.065 ± 0.071
24	Center Screen	0.523 ± 0.000	0.538 ± 0.000
25	Movement	0.052 ± 0.004	0.016 ± 0.003
26	Social Scene	0.475 ± 0.110	0.443 ± 0.159
27	Geometric Scene	0.475 ± 0.110	0.443 ± 0.159
28	Center Scene	0.538 ± 0.000	0.536 ± 0.000

The main differences between the current and the previous stimuli were fewer people in the social scenes, more vibrant colors in the geometric scenes, and a lower movement rate between frames in the current.

#### Experimental setup

First, we designed a test to evaluate whether the trained classifier from the previous study [11] was generalist enough to classify new samples with the new stimulus. For that, the trained VAMs from the previous study, trained with the previous stimulus from the reference study, were used to generate saliency maps from frames of the new stimulus, described in section “Apparatus and stimulus”. The new video was also used to create the fixation maps for each individual in the test.

To replicate the approach, we retrained the VAMs with samples collected with the new visual stimulus. Therefore, the saliency maps were built for the same video used for VAMs training. In all steps, we used the same hyperparameters of the previous study, both for the extraction and feature selection processes, as well as for classifier induction algorithms. The classifier induction algorithms used were NN and SVM with three feature sets: 1 - All, considering all 28 extracted features; 2 - Fixed, the same set of features that achieved the best result in Oliveira et al. [11]; 3 - Relief, where features with relevance greater than the average weight of all considered features by the ReliefF selector; 4 - Genetic Algorithm (GA), where the number of features considered was 15, empirically found as in the previous study. In all situations, the classifier performance was estimated using 5-fold cross-validation.

### Improvement proposal

In addition to the replication study previously presented, we designed an experiment to test a new classifier induction algorithm using a dataset previously published by the group and the the current dataset (described in sections “Subjects” and “Apparatus and stimulus”).

The NN and SVM classifier induction algorithms were presented by Oliveira et al. [11] with the ReliefF and GA feature selector algorithms. For GA, the number of selected features was 15 and the other parameters were default in the “GA_feature_selector” function. For Relief, all features with weight greater than the average of the other features were selected and k = 60 was adopted for the number of nearest neighbors in the “relief” function. Now, RF was tested with all features. Were not used feature selector algorithms, as there is already an internal feature selection in RF. To build the classifier, the number of tested trees was 25, 50, 100, and 200. To parameterize mtry (number of predictors that are randomly sampled at each split when creating the tree models), a grid search for mtry with values from 3 to 9 with step 1 was adopted. Other parameters were the default for the “TreeBagger” function [34].

#### Previous dataset

Gaze position signals were collected using a Tobii Pro TX300 [29] recording at 300 Hertz. The capture software was the Tobii Studio [35] (currently discontinued), with the use of the I-VT fixation filter [31]. Data from 106 subjects were collected to develop the model: 30 from the TD group, and 76 from the ASD group. All participants have ages ranging from three to 18 years old. The stimulus used was a video 54-second long, with no audio accompaniment, with 30 frames per second presentation. The subjects and stimulus were described in detail by Oliveira et al. [11].

#### Frame aggregation thresholds experiment

To investigate the influence of the frame aggregation threshold on the performance of the classifier for the current dataset, some thresholds were tested with RF. The threshold values were from 0.05 to 0.35 with step 0.05. In addition, we tested two other values: 0.08 (threshold where the aggregation result generates the same number of groups of frames used in the reference study) and 0.33 (threshold established in the previous study).

### Statistical analysis

Data were analyzed in RStudio version 1.3.1093 [36]. Normality and homogeneity were verified by Shapiro-Wilk and Levene’s tests, respectively. For the sample characterization data, the statistical significance of the differences in the means of groups was determined by two-tailed T-test. When assumptions were not met, the Mann-Whitney U-test was adopted. For AUC data, the statistical significance of the differences in the means of groups was determined by Wilcoxon paired test, when the compared conditions were based on the same dataset. When the dataset was different, the Mann-Whitney U-test was adopted. *P*-values < 0.05 were considered significant in all cases.

## Results

Table 2 shows the sample characterization. Regarding age comparisons, there was no statistical difference (*p*=0.89 in T-test) between TD and ASD groups from the current dataset. The comparison between the ASD groups from the current and the previous studies showed a difference in age (*p*<0.01 in T-test), with subjects 2.06 years younger in this study. The main variable for comparison between the ASD groups was the CARS score, which showed no statistical difference between the groups (*p*$=$0.21 in U-test), suggesting that the ASD severity is comparable to the previous work. Corroborating with CARS, IQ and Vineland showed no difference between the ASD groups. As expected, the female population is underrepresented in the sample, as the prevalence of ASD is higher among males [37, 38].

**Table 2:** Sample characterization. The values are expressed as average ± standard deviation. Abbreviations: TD, Typical Development; ASD, Autism Spectrum Disorder; CARS, Childhood Autism Rating Scale; IQ, Intelligence Quotient; VC, Vineland Communication; VDL, Vineland Daily Living; VS, Vineland Socialization; VL, Vineland Locomotion

Study	Current		Previous	
Groups	TD	ASD	TD	ASD
**Number**	44	33	30	76
**Age (years)**	6.7 ± 3.0	6.8 ± 3.7	9.8 ± 2.8	8.8 ± 3.7
**Male/Female**	40/4	29/4	20/10	49/27
**CARS**	-	33.9 ± 05.1	-	35.0 ± 04.0
**IQ**	104.7± 16.4	91.1 ± 15.0	-	89.0 ± 27.1
**VDL**	93.0 ± 14.4	68.1 ± 05.8	-	60.5 ± 10.7
**VS**	94.8 ± 15.6	63.2 ± 10.7	-	61.0 ± 12.6
**VC**	91.9 ± 16.3	64.3 ± 12.3	-	60.6 ± 13.4
**VL**	88.2 ± 11.3	74.8 ± 05.5	-	77.0 ± 15.5

The performance of the NN-based classifiers tested in the two scenarios, the performance of the new RF-based classifiers as well as the performance obtained in Oliveira et al. [11] are presented in Table 3. The trained classifier, that presented an average AUC of 0.82 in Oliveira et al. [11], presented an AUC value of 0.56 when applied on the current dataset, a very low value, close to an arbitrary classification, not allowing the test with a different stimulus than the one used in the training stage. On the other hand, the results of the replication experiment achieved an average AUC of 0.71, using the NN classifier induction algorithm and fixed features set (selected in the previous study). The results achieved by using different feature sets are shown in Table 4. The selected features are described in Supplementary Table 3. Therefore, the results obtained in the replication experiment were still inferior (p = 0.012 in U-test) to those achieved in the previous study but superior to the results achieved only applying the trained classifier.

**Table 3:** Performance comparison of classifiers. The values are expressed as average, except the values referring to the trained classifier. Abbreviations: AUC, Area Under the ROC Curve; Sens., Sensitivity. Spec., Specificity; NN, Neural Networks

Dataset	Approach	AUC	F1-Score	Accuracy	Sens.	Precision	Spec.
**Current**	Trained (NN)	0.56	0.53	0.58	0.55	0.51	0.61
	Replication (NN)	0.71	0.55	0.66	0.62	0.56	0.73
	Random Forest	0.74	0.64	0.67	0.67	0.61	0.67
**Previous**	Reference (NN)^a^	0.82	0.62	0.76	0.69	0.90	0.93
	Random Forest	0.95	0.91	0.88	0.88	0.95	0.87

^a^ result described by Oliveira et al., 2021, added for comparison

**Table 4:** AUCs obtained in replication experiment. The values are expressed as average ± standard deviation. Abbreviations: NN, Neural Networks; SVM, Support Vector Machine

Feature Selection	NN	SVM
All	0.67 ± 0.068	0.60 ± 0.028
Fixed^a^	0.71 ± 0.031	0.54 ± 0.046
Relief	0.61 ± 0.064	0.55 ± 0.105
Genetic Algorithm	0.64 ± 0.083	0.55 ± 0.074

^a^ represents the same features used in the best result described by Oliveira et al., 2021

The results with RF as the classifier induction algorithm achieved an average AUC of 0.74 (mtry = 9 and trees = 200) for the current dataset and 0.95 (mtry = 9 and trees = 50) for the previous dataset, as shown in Table 3. Although the RF presented a better performance in both cases, only in the second case was the performance statistically superior to the NN (*p* = 0.029 in Wilcoxon paired test).

In addition to the performance improvement with the use of RF, another benefit was the possibility of evaluating the relative importance of the features, which are presented in Supplementary Table 4. When considering the relative importance of the features to predict whether or not a pixel was fixed for each group of individuals (TD and ASD), we found that the center features (center of the screen and center of the scene) were the most important for both datasets. However, there was no difference in importance between the groups. Despite containing fewer people in the current video compared to the previous one, these features seem to have contributed similarly to the classifiers. However, the presence of face and people features had lower relative importance than the other features.

The performance obtained with the aggregation threshold (0.33) between frames used in the reference study [11] was lower for the current dataset, compared to the previous dataset. However, the movement feature presents smaller values (Table 1), requiring the aggregation of more frames to reach the established threshold. To try to mitigate this performance loss when changing the dataset, we tested other threshold values for aggregating frames. In Supplementary Fig. 1 we present the results with the variation of the threshold values. We notice an instability in the AUC values when we consider threshold values smaller than 0.2, with a tendency of performance stabilization after this threshold. The maximum value obtained was with a threshold of 0.08, where the average AUC was 0.96. Therefore, close to the better performance obtained with the previous dataset (average AUC = 0.95).

## Discussion

There was a larger imbalance between classes in the previous study, and no technique (such as SMOTE or Tomek algorithms) had been applied to overcome this. Therefore, in order to reproduce the previous study, no modification was performed in the analysis pipeline. However, in the current study, we tried to collect a less imbalanced dataset. Furthermore, we can verify that the imbalance between the groups in the previous study did not bias the classification to the majority group (ASD), since specificity was greater than sensitivity, 0.93 and 0.69, respectively.

In the trained classifier test, the results were very low when applied to a new stimulus, reaching an AUC of 0.56. Given the differences between the stimuli, even though they are based on the same paradigm, as presented in the section “Apparatus and stimulus”, this result suggests that the trained classifier cannot be used for any similar video, requiring retraining of the VAMs when changing the stimulus. In contrast, the replication experiment results with NN were superior, although inferior to those presented in the reference study. This result shows that the proposed method is robust, with potential for generalization even without reparametrization (i.e., optimize the parameters for each dataset).

As presented in the section “Apparatus and stimulus”, the main differences between the current and the previous stimuli were fewer people in the social scenes, more vibrant colors in the geometric scenes, and a lower movement rate between frames in the current. Although comorbidities may influence the results, we did not assess their influence in the results, since we did not have information about their presence in the investigated population. Despite these differences that may have impacted the performance of the classifiers with the current dataset, the robustness of the approach presented in the previous study was verified.

The results of frame aggregation threshold variation suggest that an adjustment in the movement rate is necessary to use the proposed approach. The stimulus change allows the verification of the robustness of the method, that is, the ability of the system to suppress sources of variation [39]. Accordingly, it was feasible to use a similar video to the one presented by Oliveira et al. [11], as long as the VAMs are retrained.

It is also important to note that the sample size was 27% smaller, and they are younger. However, we consider that the age difference in the ASD group between the studies was not a limitation, as the paradigm used is not age-specific and the CARS score, IQ and Vineland showed no statistical difference. This suggests the possibility of using the approach with a different sample, which can be extended to other populations.

Oliveira et al. [11] proposed a method with good results (average AUC = 0.82) without the need to demarcate ROIs and giving the opportunity to also evaluate image characteristics. Now we tested the approach with another classifier induction algorithm, the RF, which presented superior results (average AUC = 0.95) than those obtained with NN and SVM algorithms. When considering the current dataset, the results were also better, demonstrating the superiority of performance brought by the proposal. Perhaps the performance improvement is due to the internal selection of RF features, enhancing the combination of features. Also, four of the features are categorical, which is an advantage for algorithms based on decision trees. Considering that similar studies [40–42] use different approaches, methods, population, and evaluation metrics, a direct comparison with the literature is not possible. However, a closer comparison is possible with Startsev et al. [23] that, although with a different approach and static stimuli, based on face images, used RF to classify individuals with ASD. The dataset was composed of 14 TD sujects and 14 ASD subjects. They achieved an average AUC of 75% and our results obtained an average AUC of 74%. In our case, the relative importance of the face feature was low for both datasets and with values below the average of other features (Supplementary Table 4). Although the video contains fewer people, the area occupied by faces in the image was larger, as shown in Table 1.

It is widely demonstrated in the literature that center bias is important for predicting fixations, both in TD [43, 44] and ASD [10, 45]. However, in the paradigm used here, the screen is divided in half, with two scenes being displayed simultaneously. The results presented show that, in addition to the center of the screen, the center of the scenes is also important for both groups. It is important to mention that there are some metrics that penalize center bias [46, 47]. Here, there was no such penalty, with the features being considered indiscriminately, regardless of their nature.

We should highlight that there is a difference in relation to the contrast between the videos, represented in the intensity feature described by Itti [16], according to Table 1. In the current video, there are lower values for this feature. The contrast perception occurs differently between TD and ASD, and can also vary according to age [48]. However, when comparing the relative importance of the intensity feature (Supplementary Table 4), we found that there were no significant changes between the videos.

In summary, the results of the replication experiment were satisfactory, which suggests the robustness of the approach and the VAM-based approaches feasibility to aid in ASD diagnosis. In contrast, the results of the trained classifier suggest that the stimulus change between the training and testing steps influences the classifier’s performance. The proposed method change improved the classification performance, demonstrating the feasibility of RF for building VAMs. Additional studies are encouraged to test other conditions and scenarios, including the control of possible comorbidities not considered in the present study.

### Supplementary Information

**Additional file 1.**

## Data Availability

The dataset collected in the current study is available from the corresponding author on reasonable request.

## Abbreviations

ADOS: Autism diagnostic observation schedule
ASD: Autism spectrum disorder
AUC: Area under the ROC curve
CARS: Childhood autism rating scale
DSM: Diagnostic and statistical manual of mental disorders
GA: Genetic algorithm
IQ: Intelligence quotient
NN: Neural networks
RF: Random forest
ROI: Region of interest
SON-R: Non-verbal intelligence test
SVM: Support vector machine
TD: Typical development
VAM: Visual attention model
WISC: Wechsler intelligence scale for children
