# Supplementary material for: Computer-aided autism diagnosis using visual attention models and eye-tracking: replication and improvement proposal
Source: BMC Med Inform Decis Mak. 2023 Dec 14;23:285. doi: 10.1186/s12911-023-02389-9 (PMC10722824; doi:10.1186/s12911-023-02389-9)

RESEARCH

Computer-aided autism diagnosis using visual attention models and eye-tracking: replication and improvement proposal

Felipe O. Franco[1,2*], Jessica S. Oliveira[3], Joana Portolese[2], Fernando M. Sumiya[2], Andréia F. Silva[2], Ariane Machado-Lima[3], Fatima L. S. Nunes[3] and Helena Brentani[2]

*Correspondence: franco@ime.usp.br
[1]Interunit PostGraduate Program on Bioinformatics, Institute of Mathematics and Statistics (IME), University of Sao Paulo (USP), 05508-090, Sao Paulo-SP, Brazil
[2]Department of Psychiatry, University of São Paulo's School of Medicine (FMUSP), 05403-903, São Paulo-SP, Brazil. [3]School of Arts, Sciences and Humanities (EACH), University of São Paulo (USP), 03828-000, São Paulo-SP, Brazil.

Supplementary Table 1 Clinical information of subjects from the current dataset. Age is expressed in years. Abbreviations: ID, Project Identification Number; IQ, Intelligence Quotient; VC, Vineland Communication; VDL, Vineland Daily Living; VS, Vineland Socialization; VL, Vineland Locomotion; CARS, Childhood Autism Rating Scale.

ID	Class	Sex	Age	IQ	VC	VDL	VS	VL	CARS
36	TD	M	6	80	-	-	-	-	-
39	TD	F	3	93	82	85	94	83	-
56	TD	M	5	117	103	100	100	89	-
73	TD	M	13	92	85	92	66	-	-
103	TD	M	4	100	77	90	87	85	-
108	TD	M	3	105	96	79	66	89	-
127	TD	M	10	119	-	-	-	-	-
153	TD	M	5	103	77	95	87	96	-
185	TD	M	3	80	70	74	70	76	-
198	TD	M	9	114	-	-	-	-	-
210	TD	M	7	-	96	79	103	78	-
225	TD	M	4	83	-	-	-	-	-
227	TD	F	7	128	129	116	123	105	-
228	TD	F	8	120	115	110	122	102	-
259	TD	M	8	96	96	114	101	118	-
261	TD	M	8	116	67	78	87	71	-
264	TD	M	7	114	-	-	-	-	-
271	TD	M	7	120	-	-	-	-	-
278	TD	M	8	128	-	-	-	-	-
304	TD	M	10	88	76	85	87	-	-
305	TD	M	4	113	63	73	76	65	-
315	TD	M	7	85	105	110	94	85	-
316	TD	M	10	111	115	104	100	78	-
332	TD	M	12	98	96	108	110	-	-
333	TD	M	12	-	-	-	-	-	-
334	TD	M	8	118	86	90	81	85	-
336	TD	M	7	83	80	84	89	96	-
337	TD	M	11	78	115	126	122	-	-
338	TD	M	12	128	113	106	103	-	-
344	TD	M	5	-	-	-	-	-	-
347	TD	M	4	-	86	78	82	96	-
349	TD	M	5	-	79	87	98	85	-
350	TD	M	5	-	77	78	82	92	-
351	TD	M	5	-	-	-	-	-	-
352	TD	M	5	-	-	-	-	-	-
359	TD	M	4	-	81	87	88	83	-
361	TD	M	4	-	100	76	98	83	-
363	TD	M	3	-	98	90	96	89	-
369	TD	M	4	121	-	-	-	-	-
380	TD	M	4	-	88	87	115	92	-
387	TD	M	8	-	100	98	105	96	-
394	TD	M	4	-	-	-	-	-	-
396	TD	M	3	-	-	-	-	-	-
449	TD	F	13	-	107	110	112	-	-
18	ASD	M	3	95	71	73	62	83	37.5
23	ASD	M	7	100	-	-	-	-	30.5
24	ASD	M	7	-	-	-	-	-	39
25	ASD	M	4	71	51	68	64	83	31.5
28	ASD	M	6	88	-	-	-	-	31
30	ASD	M	8	-	-	-	-	-	30
38	ASD	M	4	74	-	-	-	-	44
48	ASD	M	4	-	-	-	-	-	41.5
49	ASD	M	3	74	-	-	-	-	36.5
57	ASD	M	5	80	70	68	54	68	34.5
67	ASD	M	4	-	-	-	-	-	37.5
79	ASD	M	3	86	61	62	50	73	42.5
82	ASD	M	12	-	-	-	-	-	32
83	ASD	F	3	82	47	59	56	71	41.5
84	ASD	F	3	85	43	60	52	70	38.5
101	ASD	F	10	-	-	-	-	-	33.5
102	ASD	M	5	114	76	76	82	83	37
140	ASD	M	4	-	61	68	70	74	-
141	ASD	M	4	90	76	76	70	74	30
150	ASD	M	10	110	-	-	-	-	28
156	ASD	F	15	81	-	-	-	-	28
157	ASD	M	3	91	55	74	66	73	38
160	ASD	M	6	-	-	-	-	-	-
161	ASD	M	6	81	-	-	-	-	-
184	ASD	M	8	110	-	-	-	-	26.5
201	ASD	M	12	-	81	65	76	-	28.5
202	ASD	M	7	82	-	-	-	-	-
215	ASD	M	8	-	76	65	72	71	30
236	ASD	M	5	73	-	-	-	-	31
241	ASD	M	16	122	-	-	-	-	28
242	ASD	M	5	-	-	-	-	-	30
272	ASD	M	14	107	68	72	48	-	-
381	ASD	M	10	109	-	-	-	-	-

Supplementary Table 2 Information about the stimuli.

**Stimulus**	**Previous**	**Current**
**Duration (seconds)**	53	41
**Number of frames**	1604	1242
**Resolution**	1920x1080	640x480
**Frames Per Second**	30	30

Supplementary Table 3 Features used to train classifiers based on Neural Networks. Abbreviations: TD, Typical Development; ASD, Autism Spectrum Disorder.

Dataset	Previous		Current	
Group	TD	ASD	TD	ASD
Steerable pyramids	4	3	5	5
Saliency toolbox: color, intensity, orientation and skin	4	4	2	3
RGB color	1	0	1	1
Horizon line	1	1	1	1
Presence of face	1	1	0	0
Presence of people	1	1	1	0
Distance to the frame center	0	1	1	1
Motion value	1	1	1	1
Presence of biological movement	0	1	1	1
Presence of geometrical movement	1	1	1	1
Distance to the side-specific scene center	1	1	1	1

Supplementary Table 4 Feature importance calculated by Random Forest. Abbreviations: TD, Typical Development; ASD, Autism Spectrum Disorder.

Dataset	Previous		Current	
Group	TD	ASD	TD	ASD
Steerable Pyramid	0.80	0.61	0.83	0.82
Steerable Pyramid	0.51	0.54	0.62	0.77
Steerable Pyramid	0.64	0.63	0.92	0.93
Steerable Pyramid	0.73	0.76	0.85	0.68
Steerable Pyramid	0.68	0.73	0.55	0.68
Steerable Pyramid	0.64	0.68	0.92	1.04
Steerable Pyramid	1.00	0.83	1.05	1.03
Steerable Pyramid	0.99	0.99	0.68	0.62
Steerable Pyramid	1.12	1.16	0.69	0.97
Steerable Pyramid	0.94	0.95	1.20	1.14
Steerable Pyramid	1.51	1.04	1.26	1.20
Steerable Pyramid	1.27	1.45	0.80	0.89
Steerable Pyramid	1.43	1.14	1.05	1.32
Itti Color	0.67	0.47	0.54	0.54
Intensity	0.97	0.98	0.59	0.45
Orientation	0.99	1.10	0.73	0.50
Presence of Skin	0.78	0.67	0.94	1.00
Color - Red	0.98	0.87	1.04	0.93
Color - Green	0.71	0.88	0.86	0.92
Color - Blue	0.80	0.82	0.91	0.83
Horizon Line	1.95	2.36	1.17	0.87
Presence of Face	0.52	0.52	0.58	0.56
Presence of People	0.49	0.40	0.39	0.51
Center Screen	2.82	2.69	4.22	4.41
Movement	1.15	1.34	1.07	1.08
Biological Scene	0.27	0.32	0.54	0.46
Geometric Scene	0.25	0.32	0.52	0.46
Center Scene	2.38	2.76	2.48	2.39

Supplementary Figure 1 Classification Performance According to Frame Aggregation Thresholds.

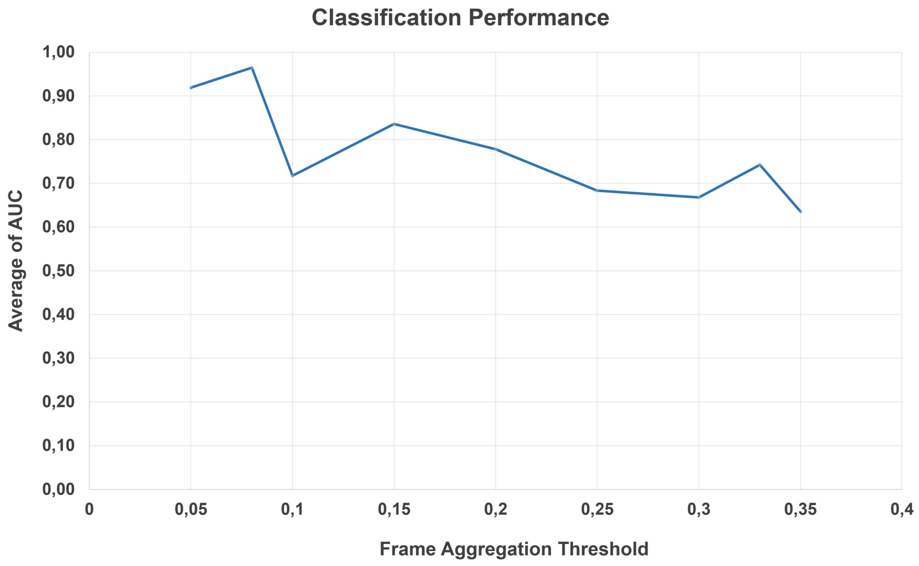

Supplement: Supplementary file 1 — Additional file 1. [file 12911_2023_2389_MOESM1_ESM.pdf]
